# A Case of Necrotic Skin Lesions on the Abdomen

**DOI:** 10.5811/cpcem.2018.9.40042

**Published:** 2018-09-28

**Authors:** Benjamin Christians, Dana King

**Affiliations:** University of Iowa, Department of Emergency Medicine, Iowa City, Iowa

## CASE PRESENTATION

**Image 1:**
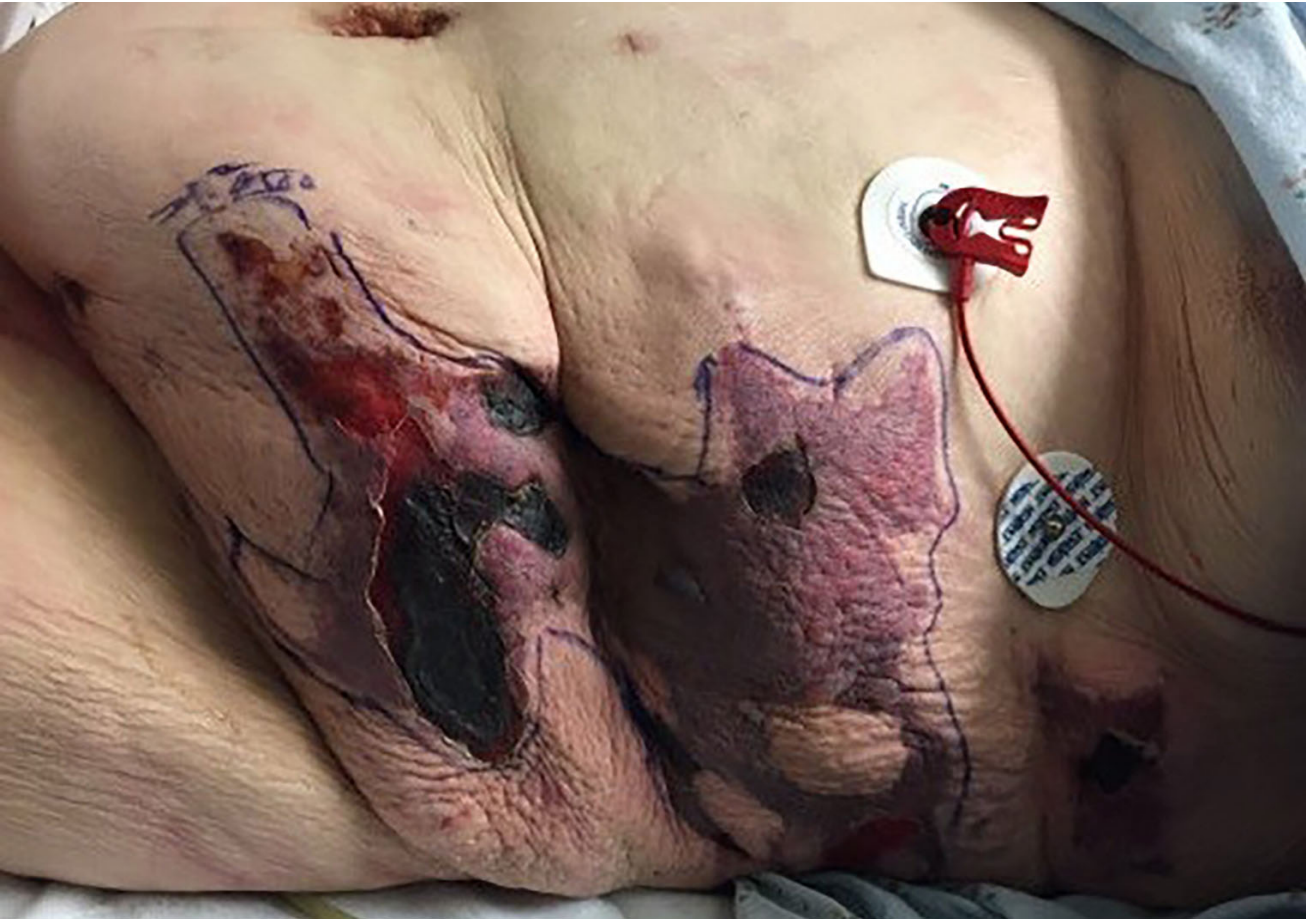
Photo of patient’s left lower abdomen and flank showing areas of erythema and centralized necrotic lesions representative of calciphylaxis outlined by surgical marking pen.

**Image 2:**
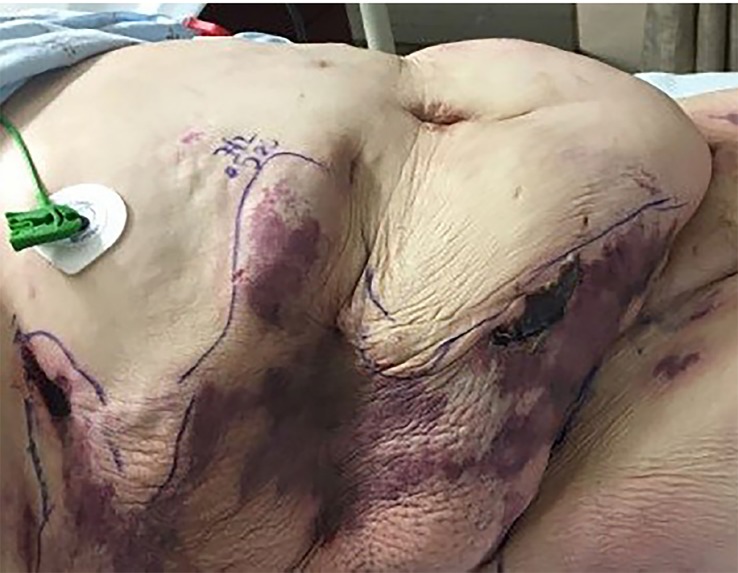
Photo of patient’s right lower abdomen and flank showing areas of erythema and centralized necrotic lesions representative of calciphylaxis outlined by surgical marking pen.

A 42-year-old female with a history of type II diabetes, partial left nephrectomy, and fibromyalgia was transferred from an outside hospital for concerns of a painful rash on her abdomen and flanks. She was admitted for sepsis and acute kidney injury at the outside hospital three weeks prior to arrival, and was discharged one week later on subcutaneous enoxaparin for deep vein thrombosis prophylaxis. She noticed bruising and rash to her bilateral lower abdomen one week after discharge with progressive pain. She presented to an outside emergency department (ED) for rash and pain control. Abdominal computed tomography showed diffuse body wall edema with no subcutaneous air. The local consulting surgeon did not believe the patient had necrotizing fasciitis but was unsure of diagnosis of the rash. She received piperacillin/tazobactam, vancomycin, and one unit of packed red blood cells prior to transfer. Upon arrival to our ED, physical exam showed tender necrotic firm lesions to her bilateral lower abdomen and flanks with surrounding erythema (Images 1 and 2).

## DISCUSSION

### Diagnosis: Non-uremic Calciphylaxis

Calciphylaxis is a rare, life-threatening vascular syndrome characterized by calcification of microvasculature causing thrombosis and resultant soft tissue ischemia and necrosis. Diagnosis is made clinically, supported by skin biopsy demonstrating arterial calcifications and occlusions without vasculitis. It is most commonly seen in patients with end-stage renal disease or imbalance in calcium homeostasis with an incidence of 35 cases per 10,000 patients.^1^ In a review of 36 cases of nonuremic calciphylaxis, the majority of patients were found to have hyperparathyroidism or recent glucocorticoid use as a risk factor.^2^,^3^ Consequently, its occurrence in our patient, who had no known risk factors for nonuremic calciphylaxis, was an extremely rare event.

In consultation with general surgery and dermatology, we concluded that infectious or vasculitic etiologies were the most likely causes. A wound-edge biopsy demonstrated areas of vascular calcification, intimal hyperplasia, and scattered soft tissue calcinosis consistent with calciphylaxis. Dermatology recommended treatment of the lesions with sodium thiosulfate, 0.25% acetic acid irrigation, and collagenase ointment. She was admitted and completed a course of vancomycin and ceftriaxone and subsequently was discharged home without complications.

**CPC-EM Capsule**

What do we already know about this clinical entity?

Literature has described calciphylaxis frequently in patients receiving dialysis or with uremia, and it is associated with high mortality.

What is the major impact of the image(s)?

These images, which show the visual appearance of calciphylaxis in a patient without end-stage renal disease, will aid in the recognition of this entity and its inclusion in differential diagnosis.

How might this improve emergency medicine practice?

This is a rare presentation that mimics other life-threatening conditions. Greater awareness of calciphylaxis will lead to earlier diagnosis, consultation and treatment, as outcomes are historically poor.

Documented patient informed consent and/or Institutional Review Board approval has been obtained and filed for publication of this case report.
